# Inhibition of Calpain Prevents Manganese-Induced Cell Injury and Alpha-Synuclein Oligomerization in Organotypic Brain Slice Cultures

**DOI:** 10.1371/journal.pone.0119205

**Published:** 2015-03-10

**Authors:** Bin Xu, Wei Liu, Yu Deng, Tian-Yao Yang, Shu Feng, Zhao-Fa Xu

**Affiliations:** Department of Environmental Health, School of Public Health, China Medical University, Shenyang, Liaoning, People’s Republic of China; Martin Luther University, GERMANY

## Abstract

Overexposure to manganese has been known to promote alpha-synuclein oligomerization and enhance cellular toxicity. However, the exact mechanism of Mn-induced alpha-synuclein oligomerization is unclear. To explore whether alpha-synuclein oligomerization was associated with the cleavage of alpha-synuclein by calpain, we made a rat brain slice model of manganism and pretreated slices with calpain inhibitor II, a cell-permeable peptide that restricts the activity of calpain. After slices were treated with 400 μM Mn for 24 h, there were significant increases in the percentage of apoptotic cells, lactate dehydrogenase release, intracellular [Ca^2+^]_i_, calpain activity, and the mRNA and protein expression of calpain 1 and alpha-synuclein. Moreover, the number of C- and N-terminal fragments of alpha-synuclein and the amount of alpha-synuclein oligomerization also increased. These results also showed that calpain inhibitor II pretreatment could reduce Mn-induced nerve cell injury and alpha-synuclein oligomerization. Additionally, there was a significant decrease in the number of C- and N-terminal fragments of alpha-synuclein in calpain inhibitor II-pretreated slices. These findings revealed that Mn induced the cleavage of alpha-synuclein protein via overactivation of calpain and subsequent alpha-synuclein oligomerization in cultured slices. Moreover, the cleavage of alpha-synuclein by calpain 1 is an important signaling event in Mn-induced alpha-synuclein oligomerization.

## Introduction

Manganese (Mn) is an essential element that functions as a cofactor for numerous homeostatic and trophic enzymes in the central nervous system (CNS). Normal Mn concentrations in human whole blood are ≤ 10–12 μg/L. But at abnormally high intake levels, Mn accumulates in the brain and causes neurotoxicity [1]. The wide use of Mn in a range of industries has led to global health concerns. Indeed, Mn intoxication occurs from occupational exposure [2], administration of total parenteral nutrition [3], and chronic liver failure [4]. Concern about Mn exposure has also focused on the use of a Mn-containing fuel additive, methylcyclopentadienyl Mn tricarbonyl (MMT), as an anti-knock agent in gasoline in Canada and other Western nations [5]. Exposure to high levels of Mn can cause neurotoxicity and also the development of a form of Parkinsonism known as manganism. It has recently been hypothesized that Mn exposure might also cause or accelerate the development of Parkinson disease (PD). In China, accumulation of Mn and Fe via unknown routes might be involved in the etiology of PD in the general population [6]. Therefore, understanding the exact molecular mechanisms of Mn neurotoxicity may play a critical role in linking environmental neurotoxins to the pathogenesis of PD.

Although oxidative stress, energy failure, and the disturbance of neurotransmitter metabolism have been actively investigated as neurotoxic mechanisms of Mn over the past two decades [7,8], emerging evidence indicates that alpha-synuclein oligomerization is also one of the important cellular and molecular correlates of neurodegenerative diseases resulting from chronic Mn exposure [9]. Alpha-synuclein is a small protein that plays an important role in synaptic plasticity, regulation of vesicle transport, and dopaminergic neurotransmission. Numerous studies now support the hypothesis that alpha-synuclein oligomerization is the key step driving pathology, cellular damage, and subsequent neuronal dysfunction [10,11]. The evidence suggests that early intermediary oligomers, rather than mature fibrils of alpha-synuclein, are the pathogenic species [12]. Alpha-synuclein overexpression promotes apoptotic cell death in a variety of cell lines and animal models [13]. We found in a previous study that manganese could induce alpha-synuclein oligomerization, leading to neuronal injury [14]. The early oligomeric intermediates are assumed to be very toxic to the cell and can induce leaking in vesicles [15]. Although the majority of the previous studies have focused on the aggregation of full-length alpha-synuclein, recent studies suggest that truncated forms of alpha-synuclein are of pathogenic significance: they promote the ability of full-length alpha-synuclein to aggregate and enhance cellular toxicity [16]. Moreover, co-expression of both full-length human alpha-synuclein and C-terminally truncated human alpha-synuclein can augment the accumulation of pathological full-length alpha-synuclein and lead to DAergic cell death [17]. The mechanisms governing the proteolytic cleavage of alpha-synuclein are not firmly established, but a potential candidate protease is calpain. Calpain 1 is one of a large family of intracellular calcium-dependent proteases whose cleavage of specific proteins has been implicated in physiological pathways and in numerous pathological diseases [18]. Alpha-synuclein is a substrate for calpain cleavage, and calpain cleaved alpha-synuclein species could promote alpha-synuclein aggregation and enhance cellular toxicity [19]. Thus, we speculated that calpain overactivation was one of the important pathogenic mechanisms of neurodegenerative diseases resulting from chronic Mn exposure and might play a role in alpha-synuclein oligomerization.

Although calpain overactivation contributes to neurodegeneration, calpains also serve essential physiological roles including signal transduction, cell migration, membrane fusion, and cell differentiation. Thus, the challenge is to inhibit the pathological consequences of calpain overactivation while preserving physiologic aspects of calpain function. Calpain inhibitor II, a cell-permeable peptide that restricts the activity of calpain, has been shown to successfully prevent methylmercury from causing neuronal cell death [20], but it was still unclear whether calpain inhibitor II would prevent Mn-induced alpha-synuclein oligomerization. Therefore, to verify our hypothesis, calpain inhibitor II was used in this study. We present data showing that cleavage of alpha-synuclein by calpain occurs in Mn-treated brain slices. Furthermore, calpain inhibition II attenuated the alpha-synuclein oligomerization that occurred as a result of Mn treatment.

## Material and Methods

### Ethics statement

The animal experiment was carried out according to the National Institutes of Health Guidelines for the Care and Use of Laboratory Animals and approved by the Institutional Animal Care and Use Committee of China Medical University. All efforts were made to minimize the number of animals used and their suffering.

### Chemicals

Manganese (II) chloride tetrahydrate, calpain inhibitor II, and Fura-2 pentakis (acetoxymethyl) ester (Fura 2-AM) were obtained from Sigma Chemical Co. (St. Louis, MO, USA). The annexin Ⅴ-FITC/PI reagent kit was from Nanjing KeyGen Biotech. Co. Ltd. (Cat No: KGA106; China). *PrimeScript RT* Enzyme Mix I and *SYBR* Premix Ex TaqTM II kits were from TaKaRa Biotech. Co. Ltd. Polyacrylamide gradient gel (4–20%) was obtained from Dycent Biotech Co. Ltd. (Cat No: B1611105; China). Mouse β-actin primary antibodies were purchased from Santa Cruz Biotechnology, Inc. (Santa Cruz, CA). Mouse full-length alpha-synuclein monoclonal antibody (LB 509) and rabbit calpain 1 polyclonal antibody were purchased from Abcam (Hong Kong) Ltd. We also generated our own antibodies based on a previously reported strategy [21]. We chose two peptides for immunization in rabbits at GL Biochem, Ltd (Shanghai, China). KAKEGVVAA represents the neoepitope region of the fragment that would be generated after cleavage of alpha-synuclein by calpain between amino acids 9 and 10. We refer to this antibody as the N-terminal alpha-synuclein calpain cleavage product antibody (CCP Ab). We also synthesized a site directed antibody to the C-terminus after cleavage of alpha-synuclein between amino acids 122 and 123. A peptide corresponding to the upstream neoepitope fragment that would be generated after cleavage of alpha-synuclein at this site (CPVDPDN) was synthesized, conjugated to keyhole limpet hemocyanin, and injected into rabbits. We refer to this antibody as the C-terminal alpha-synuclein CCP Ab. Horseradish peroxidase (HRP) conjugated anti-rabbit secondary antibody and HRP conjugated anti-mouse secondary antibody were purchased from Abcam. Other analytical grade chemicals were obtained from local chemical suppliers.

### Preparation of organotypic slice cultures

Organotypic slice cultures were prepared according to the methods described previously [22,23]. Briefly, Wistar rats at postnatal days 3–4 were provided by the Laboratory Animal Center of China Medicine University (SPF grade, production license No. SCXK2008–0005). Neonatal rats were decapitated. After the brains were dissected and the frontal and occipital poles (including the cerebellum) were removed, the specimens were placed into Hank’s balanced salt solution (HBSS, Invitrogen) and kept at 4°C and pH 7.35. The specimens were sliced into 300 μm thick sections on a NVSL/NVSLM1 tissue slicer (World Precision Instruments Inc., USA). The first few slices (in most cases not more than four) were discarded until the typical cytoarchitecture of the basal ganglia was visible. Generally 4–6 slices with intact basal ganglia cytoarchitecture were collected, transferred to cell culture inserts (pore size 0.4 μm, Falcon, Millipore, Bedford, MA), placed in 6-well culture dishes (Falcon) and fed with 1 ml culture medium consisting of 50% minimum essential medium, 24% horse serum, 25% HBSS, and 1% penicillin-streptomycin (all from Invitrogen) and supplemented with 36 mM glucose and 25 mM Hepes (Sigma, St. Louis, MO, USA) (pH 7.2). Cultures were maintained at 37°C under room air + 5% CO_2_. After 1 day in culture, the culture medium was replaced with fresh medium containing no antibiotics.

### Drug treatment

Cultured slices at days 13–15 in vitro were incubated for 24–48 h in serum-free medium, in which minimum essential medium/Hepes was substituted for horse serum. Next, slices were exposed to Mn (0 or 400 μM) for 24 h and Calpain inhibitor II (1, 2 or 4 μM) dissolved in serum-free medium. For L-Canavanine supplementation experiments, cultured slices were maintained in these media at 37°C, 5% CO_2_ for 12 h before treatment with Mn.

### LDH release assays

After treatment, lactate dehydrogenase (LDH) release in the medium was measured using methods previously described [24]. The absorbance values were read at 440 nm using a microplate reader (Bio-Rad, USA), and the results of the absorbance from the test wells were expressed as a percentage of the control wells. Results from a single experiment are reported. Similar data were obtained in six independent experiments.

### Apoptosis assays

After 24 hours of treatment with Mn, brain slices were used for the preparation of dissociated slices cells as described by villalba [25]. Briefly, the slices were washed 3–5 times with phosphate buffered saline (PBS, pH 7.2~7.4). The slices were minced in 2 ml PBS supplemented with 0.125% trypsin for 10–15 min with vigorous shaking at 37°C. The subsequent mechanical dissociation with Pasteur pipettes and nylon mesh screens was described by Villalba et al. Cells were suspended in PBS supplemented with 0.1% bovine serum albumin (BSA) until used. The cell concentration was evaluated by viable cell count (trypan blue stained) and was diluted to 1×10^6^ cells/ml for apoptosis detection. Staining was performed according to the manufacturer’s manual. Using flow cytometry (FCM), 1×10^6^ cells per sample were analyzed. Annexin Ⅴ-FITC is a sensitive probe for identifying cells undergoing apoptosis, because phosphatidylserine (PS) exposure occurs early in the apoptotic process. PI is a nonspecific DNA dye that is excluded from living cells with intact plasma membranes but incorporated into nonviable cells, which might have been damaged when the cells were dissociated from the slices. So, the population of cells that was only positive for Annexin V (Q4 quadrant, Annexin Ⅴ-FITC＋/PI－) were considered to be the early apoptotic cells. The early apoptotic cells from Q4 were reported as a percentage of the total number of cells.

### Measurement of intracellular free calcium

After preparation of the dissociated cells, the [Ca^2+^]_i_ assay was performed by a method described previously [26]. Briefly, for fura-2 experiments, absolute values of [Ca^2+^]_i_ in the neurocyte were calibrated from the measured fluorescence signals using an F-4500 Fluorescence Spectrophotometer (HITACHI, Japan). Results of an individual experiment are reported. Similar data were obtained in six independent experiments. The calibration equation used was: [Ca^2+^]_i_ = K_d_ [(R-R_min_) / (R_max_-R)]×(S_f380_/S_b380_). [Ca^2+^]_i_ is the concentration (nM) of intracellular Ca^2+^; K_d_ is the dissociation constant of the dye; *R* is the ratio at excitation wavelengths 340/380 nm; *R*_*min*_ is the ratio at zero [Ca^2+^]_i_; and *R*_*max*_ is the ratio at saturated [Ca^2+^]_i_. The procedures for obtaining *R*_*max*_ and *R*_*min*_ caused damage to cells and were therefore performed at the end of the experiments. *R*_*max*_ was obtained first by adding Triton X-100 (0.2%), making the cell membrane permeable to Ca^2+^ and allowing the extracellular and intracellular Ca^2+^ to equilibrate. Next, *R*_*min*_ was obtained by adding the chelator EGTA [ethylene glycol bis(β-aminoethyl ether)-N, N, N′, N′-tetraacetic acid; 20 mM] to chelate all Ca^2+^ inside and outside the cells. The present experiments were carried out at pH7.4 and a temperature of 37°C. A K_d_ value of 224 nM was used. The results are expressed as a percentage of the controls.

### Measurement of calpain activity

Calpain activity was assayed as described by Buroker-Kilgore and Wang [27]. Briefly, after the brain slices were homogenized in an extraction medium containing 5 mM β-mercaptoethanol, 0.1 mM EDTA, lysocephalin 5 mM, DL-Dithiothreitol 10 mM and 20 mM Tris-HCl at pH 8.6, tissue homogenate was centrifuged at 1000 × g for 10 min to remove the protein precipitate. Samples were incubated with the calpain substrate casein, and after removal of an aliquot, Coomassie brilliant blue G-250 dye reagent was added to the aliquot and was quantified using a spectrophotometer at 595 nm. The calpain activity was calculated as the difference between samples with and without Ca^2+^. The results are expressed as a percentage of the controls.

### Quantitative real-time PCR analysis

The mRNA expression levels were analyzed using a real-time reverse-transcription polymerase chain reaction assay. Total RNA was isolated using TRIzol reagent (TaKaRa Biotech. Co. Ltd., China). The first strand cDNA was synthesized from 1 μg of total RNA by reverse transcriptase using *PrimeScript* RT Enzyme Mix I (TaKaRa Biotech. Co. Ltd., China) and oligo (dT) primers according to the manufacturer’s protocol. Real-time quantitative PCR (qPCR) was performed by *SYBR Premix* Ex TaqTM II kit (TaKaRa Biotech. Co. Ltd., China) using an ABI 7500 Real-Time PCR System (Applied Biosystems, USA). Two microliters of template cDNA was added to the final volume of 20 μl of reaction mixture. Real-time PCR cycle parameters were 30 sec at 95°C followed by 40 cycles with denaturing at 95°C for 5 sec, annealing at 60°C for 34 sec and elongating at 72°C for 20 sec. The sequences of the specific primer sets for calpain 1, alpha-synuclein and β-actin used in this study are given in Table 1 [28,29]. Expression of selected genes was normalized with the gene for β-actin, which was used as an internal housekeeping control. For relative quantification of the genes tested, we used the comparative CT method (ΔΔCT). All the real-time PCR experiments were performed in quadruplicate, and data were expressed as the mean of at least four independent experiments.

**Table 1:** Primer Sequences Used for the Amplification of Each Gene in this Study.

Name	Oligo	Primer sequence
alpha-synuclein	Sense primer	5’- CACAAGAGGGAATCCTGGAA-3’
	Anti-sense primer	5’- TCATGCTGGCCGTGAGG-3’
calpain 1	Sense primer	5’- ACCACATTTTACGAGGGCAC-3’
	Anti-sense primer	5’- GGATCTTGAACTGGGGGTTT-3’
β-actin	Sense primer	5’- GGAGATTACTGCCCTGGCTCCTA-3’
	Anti-sense primer	5’- GACTCATCGTACTCCTGCTTGCTG-3’

β-actin was used as a constitutively expressed gene, and all the data were normalized against β-actin expression.

### Western blotting analysis

Protein extraction and immunoblot analyses were conducted as described previously [30]. Brain slices were rinsed with PBS and total protein was extracted from the neurons using RIPA buffer (10 mM Na_2_HPO_4_, pH 7.2, 150 mM NaCl, 1% sodium deoxycholate, 1% Nonidet P-40, 0.1% SDS) containing protease inhibitors. Protein concentrations were determined using the BCA reagent from Pierce. Thirty micrograms of protein per sample were loaded onto a 10% polyacrylamide gel, separated by electrophoresis, and transferred to polyvinylidene difluoride (PVDF) membranes (Millipore, Ternicula, CA). PVDF membranes were blocked overnight at 4°C in TBST containing 5% bovine serum albumin fraction V. The membranes were then rinsed briefly in TBST and incubated with calpain 1 antibody, full-length alpha-synuclein antibody, C-terminal alpha-synuclein CCP Ab, N-terminal alpha-synuclein CCP Ab or β-actin primary antibody in TBST for 2 h at room temperature. Specific protein expression was then detected by incubating the washed membranes with HRP conjugated secondary antibodies. Protein bands were visualized using ECL Western blotting chemiluminescent detection reagents (Pierce) and autoradiography. The intensity of the bands was evaluated semi-quantitatively by densitometry using image analyzing software (FluorChem v2.0). The changes in intensity of protein bands after Mn treatment were normalized with the intensity obtained in the internal control bands (β-actin). Representative results from a single experiment are shown. Similar data were obtained from at least four independent experiments.

### Immunocytochemistry of alpha-synuclein and calpain 1 in neuronal cells

After 24 hours of treatment with Mn, cultured slices were fixed with 4% paraformaldehyde in 0.1 M phosphate buffer containing 4% sucrose for 2 h and were frozen subsequently. After the sections were rinsed with PBS, they were permeabilized and blocked in 0.5% Triton X-100 in PBS containing 5% donkey serum (Jackson ImmunoResearch Laboratories Inc., USA), then they were incubated with these primary antibodies overnight at 4°C: rabbit anti-C-terminal alpha-synuclein polyclonal antibody (1:50) and mouse anti-calpain 1 polyclonal antibody (1:100). After being rinsed with PBS, slices were incubated with these secondary antibodies for 2 h at room temperature: Alexa Fluor 488-labeled donkey anti-rabbit IgG and Alexa Fluor 594-labeled donkey anti-mouse IgG (1:1000; Molecular Probes, Invitrogen, Carlsbad, CA). The fluorescent signal was examined on an Olympus confocal microscope (FV 1000S- IX81, Olympus, Japan), using the 40× objective lens. Confocal laser scanning microscope digital images were collected and saved in TIFF format, using FV10-ASW software (ver. 03.00.01.15, Olympus).

### Co-immunoprecipitation (Co-IP) of alpha-synuclein with calpain 1 in neuronal cells

A Co-IP assay was conducted as described previously [31]. After 24 hours of treatment with Mn, brain slices were rinsed three times in ice-cold PBS buffer, followed by incubation for 30 min at 4°C with 1 ml ice-cold IP lysis buffer (Beyotime, Haimen, China) containing the serine protease inhibitor phenylmethanesulfonyl fluoride (PMSF). Samples were pre-cleared with Protein A+G agarose (Beyotime). Pre-cleared lysates were then incubated with mouse anti-full-length alpha-synuclein monoclonal antibody (1:50) or rabbit anti-calpain 1 polyclonal antibody (1:50) at 4°C overnight. A 25% slurry of Protein A+G agarose was added into the lysates incubated for 2 h at 4°C and washed with ice-cold IP lysis buffer (Beyotime). The pellet was resuspended in SDS loading buffer, boiled for 10 min, and then centrifuged at 12,000 × g for 1 min in a Sigma 3K 30 centrifuge (Sigma, Germany). The supernatant was removed and loaded onto a 12% SDS-PAGE gel separated by electrophoresis and transferred onto a PVDF membrane. Membrane blots were probed with rabbit anti-calpain 1 polyclonal antibody or mouse anti-full-length alpha-synuclein monoclonal antibody and visualized by chemiluminescent detection reagents (Pierce).

### Alpha-synuclein oligomerization assay

The intracellular alpha-synuclein oligomers were measured as described in our previous study [14]. Briefly, after total protein was extracted, protein concentrations were determined with the BCA reagent (Pierce). Equal amounts of protein from each fraction were mixed with 2× non-denaturing protein loading buffer [without sodium dodecyl sulfate (SDS) and DL-Dithiothreitol (DTT)], without boiling, loaded onto a 4–20% nondenaturing polyacrylamide gradient gel (Dycent Biotech Co. Ltd., China), electrophoresed and transferred to PVDF membranes (Immobilon-P^SQ^, Millipore). PVDF membranes were blocked overnight at 4°C in TBST containing 5% BSA. Membrane blots were probed with full-length alpha-synuclein and mouse anti-β-actin monoclonal antibody and visualized by chemiluminescent detection reagents (Pierce). The change in the intensity of alpha-synuclein protein after Mn treatment was normalized with the intensity obtained from the internal control bands (β-actin). Representative results of an individual experiment are shown. Similar data were obtained from at least four independent experiments.

### Statistical analysis

Statistical analyses were performed using SPSS 11.0 and the results were expressed as the mean±S.D. Differences between the means were determined by one-way ANOVA followed by a Student—Newman—Keuls test for multiple comparisons. Differences at either *P*<0.05 or *P*<0.01 were considered statistically significant.

## Results

### Mn-induced nerve cell injury in brain slices could be ameliorated by calpain inhibitor II

**Fig 1:**
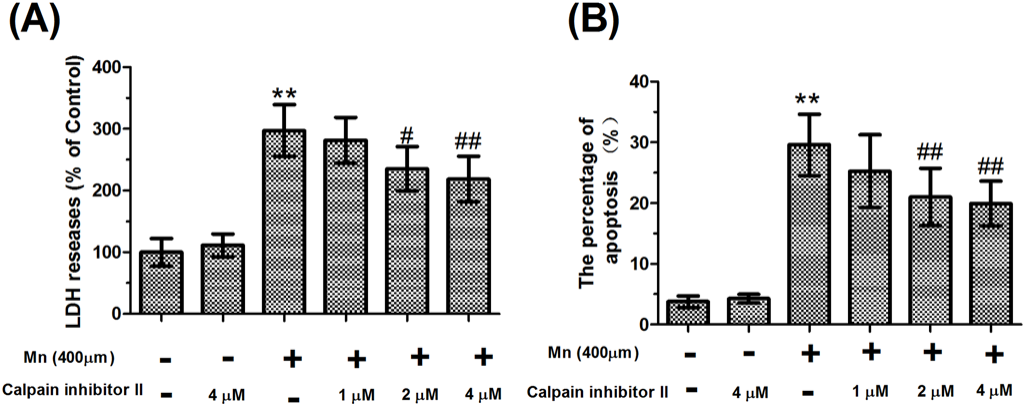
Mn-induced nerve cell injury in brain slices could be ameliorated by calpain inhibitor II. (A) After 24 hours of treatment with Mn, the increase in LDH release in culture medium, as measured using a microplate reader at 440 nm, was prevented by calpain inhibitor II. (B) Dissociated cells were analyzed using flow cytometry. Q4 quadrant positive cells (FITC＋/PI－), which are shown in S1 Fig., were considered to be the early apoptotic cells. Data are expressed as a percentage of controls, with the mean±S.D. from six different experiments. ** *P*<0.01 compared with control slices; ^*#*^ *P*<0.05 and ^# #^ *P*<0.01, compared with 400 μM Mn-treated slices.

To estimate the nerve cell injury in a brain slice, we measured the LDH released into the culture medium and the percentage of early apoptotic cells from brain slices, after the brain slices had been treated with Mn for 24 hours. LDH release is an indicator of the integrity of the cell membrane because LDH is released from cells after the cells are injured. Slices treated with 400 μM Mn released significantly more LDH into the culture medium (2.97-fold of control, *P*<0.01) indicative of overt cytotoxicity. Interestingly, the levels of LDH released from the slices pretreated with calpain inhibitor II (2 and 4 μM) were significantly decreased compared with the levels from the slices only treated with 400 μM Mn (20.86% and 26.45%, respectively, *P*<0.05, Fig. 1A), but the levels of LDH released were still higher than those in the absolute controls. The percentage of early apoptotic cells was determined by flow cytometry using double staining with Annexin-V-FITC/PI, after the slices had been treated with 400 μM Mn. Treatment of the slices with 400 μM Mn resulted in a significant increase in the percentage of early apoptotic cells (7.85-fold of control, *P*<0.01). However, pretreatment with calpain inhibitor II (2 and 4 μM) reduced Mn-induced cell apoptosis, which were still higher than those in the absolute controls (Fig. 1B). These data suggest that calpain inhibitor II pretreatment could reduce Mn-induced nerve cell injury, and there were no obvious differences in neurotoxicity of the slices treated with 4 μM calpain inhibitor II alone compared with controls.

### Calpain inhibitor II abated the Mn-induced overactivation of calpain

**Fig 2:**
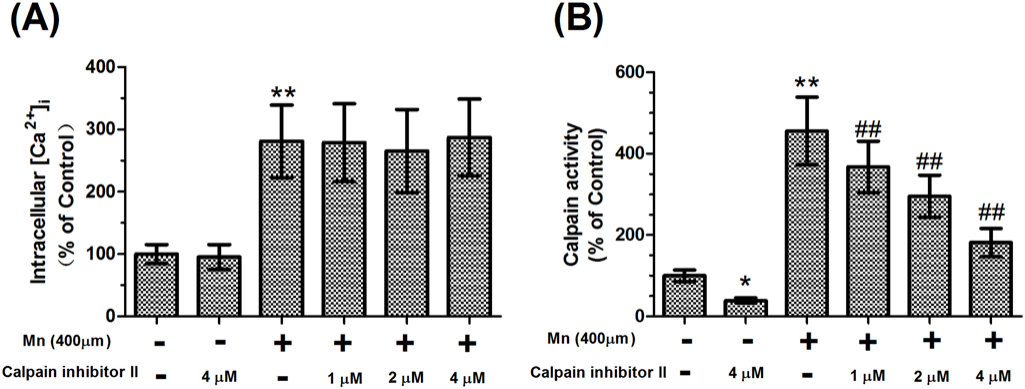
Effects of Mn and calpain inhibitor II pretreatment on intracellular Ca^2+^ and calpain activity in cultured slices. (A) [Ca^2+^]_i_ in the dissociated cells was calibrated from the measured fluorescence signals by the use of an F-4500 Fluorescence Spectrophotometer. (B) After brain slices were homogenized, calpain activity was measured using a spectrophotometer at 595 nm. Data are expressed as a percentage of controls, with the mean±S.D. from six different experiments. * *P*<0.05 and ** *P*<0.01 compared with control slices; ^# #^ *P*<0.01, compared with 400 μM Mn-treated slices.

Calpains are intracellular calcium-dependent proteases. To examine whether Mn affects calpain activity, we first measured the change of intracellular [Ca^2+^] using the Ca^2+^ indicator Fura-2 AM. Treatment of brain slices in vitro with Mn for 24 hours resulted in a significant increase in [Ca^2+^]_i_ (2.81-fold of control, *P*<0.01, Fig. 2A). [Ca^2+^]_i_ was not significantly different in slices pretreated with calpain inhibitor II compared with 400 μM Mn-treated slices, which were still higher than those in the absolute controls (Fig. 2A). Similar to the increase in [Ca^2+^]_i_ produced by treatment with Mn, treatment with Mn also caused a significant increase in calpain activity compared with controls (4.56-fold of control, *P*<0.01, Fig. 2B). However, calpain activity decreased 60.66% in slices treated with 4 μM calpain inhibitor II alone compared with the controls (*P*<0.05). Pretreatment with calpain inhibitor II caused a dose-dependent decrease in calpain activity, with the maximum decrease in the slices pretreated with 4 μM calpain inhibitor II (60.10%, *P*<0.01, Fig. 2B), but calpain activity was still higher than those in the absolute controls. These data suggested that calpain inhibitor II could inhibit the activity of calpains. However, there was no effect on [Ca^2+^]_i_.

### Mn promoted mRNA and protein expression of calpain 1 and alpha-synuclein

**Fig 3:**
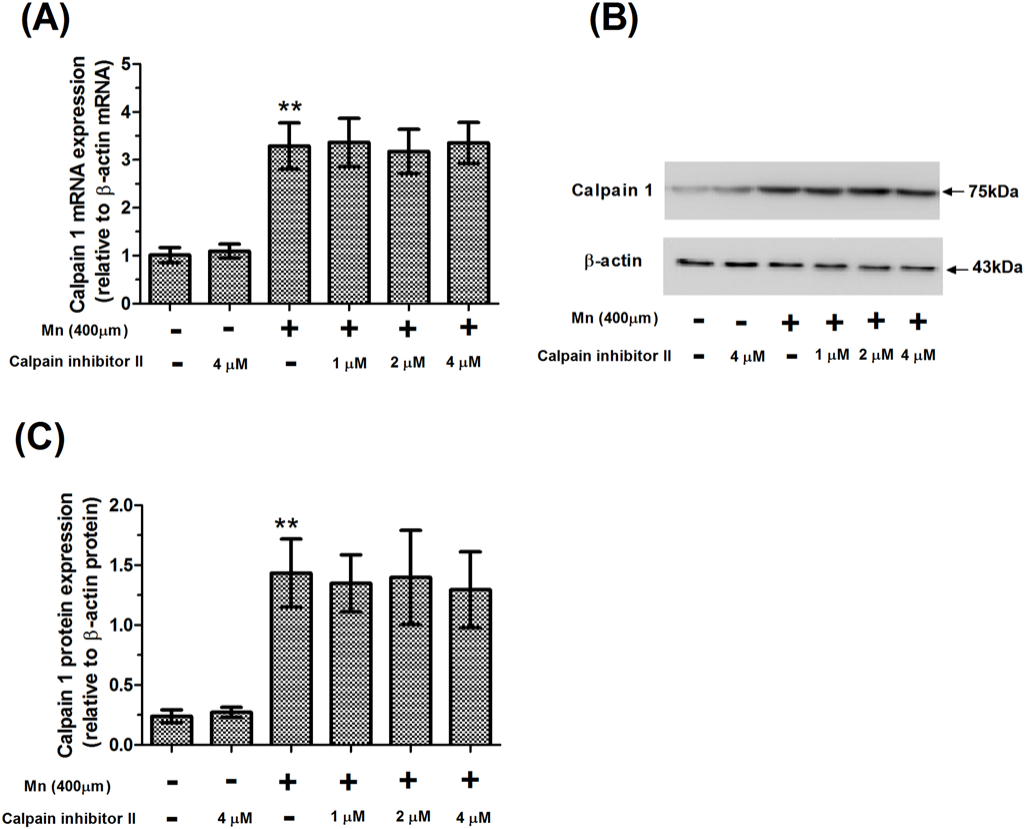
Mn promoted calpain 1 mRNA and protein expression. After brain slices were treated with calpain inhibitor II and Mn, mRNA and protein expression of calpain 1 were measured from brain homogenates. (A) The mRNA expression levels were analyzed using a real-time qPCR assay. Expression of the calpain 1 gene was normalized with β-actin gene expression, using the comparative CT method (ΔΔCT). (B) Western blotting for calpain 1 and β-actin in the calpain inhibitor II and Mn-treated slices. (C) Semi-quantitative analysis of the protein expression of calpain 1. Expression of calpain 1 protein was normalized with β-actin protein. ** *P*<0.01 compared with control slices.

We also examined the effect of Mn on the mRNA and protein expression of calpain 1 in cultured slices. As shown in Fig. 3, we found that the mRNA and protein expression of calpain 1 significantly increased in 400 μM Mn-treated slices (3.26 and 6.01-fold of control, respectively, *P*<0.01), and pretreatment with calpain inhibitor II had no effect on the mRNA and protein expression of calpain 1 when compared to 400 μM Mn-treated slices. Pretreatment with calpain inhibitor II also caused a significant increase in the mRNA and protein expression of calpain 1 when compared to the absolute controls.

**Fig 4:**
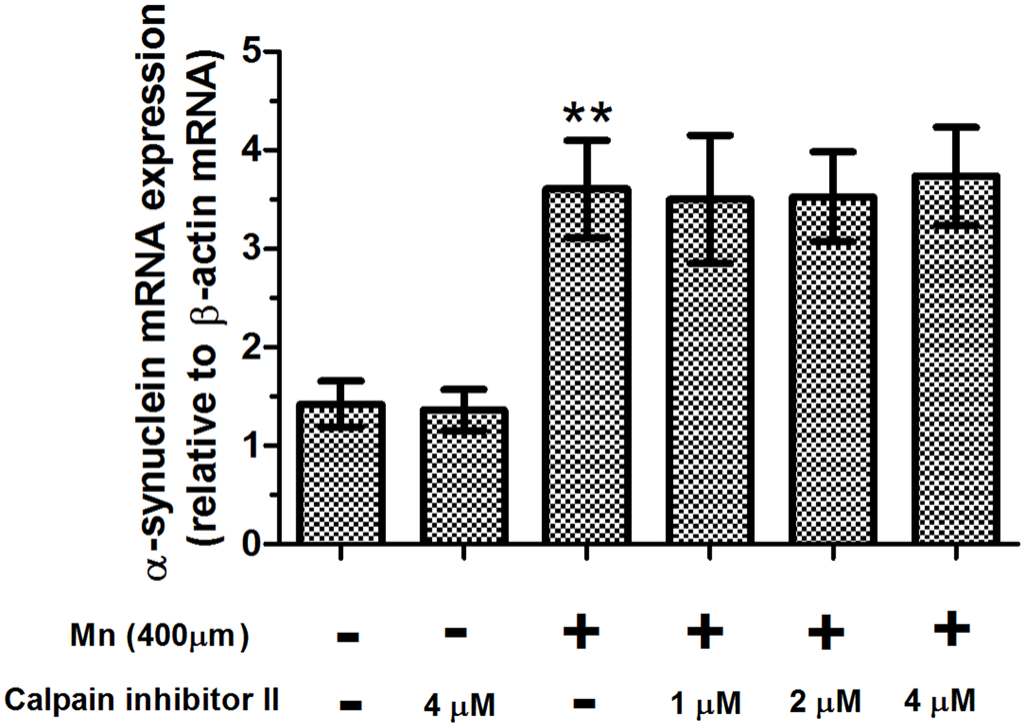
Mn promoted alpha-synuclein mRNA expression. After brain slices were treated with calpain inhibitor II and Mn, mRNA expression of alpha-synuclein was measured from brain homogenates. The mRNA expression levels were analyzed using a real-time qPCR assay. Expression of alpha-synuclein gene was normalized with β-actin gene expression, using the comparative CT method (ΔΔCT). ** *P*<0.01 compared with control slices.

**Fig 5:**
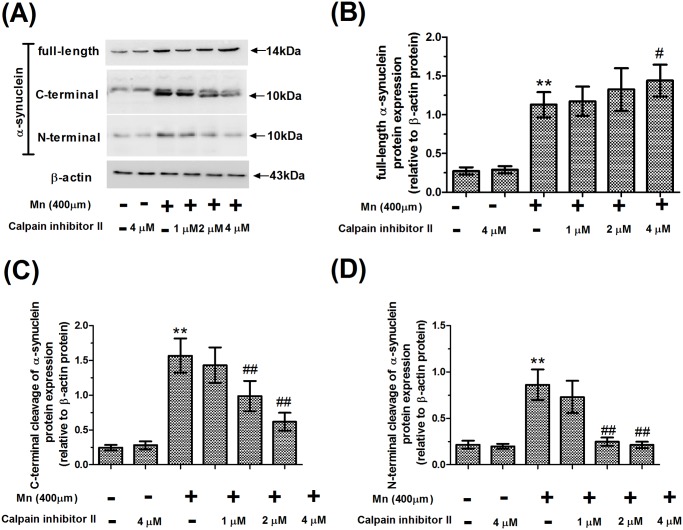
Cleavage of alpha-synuclein occurs in Mn-treated slices. After brain slices were treated with calpain inhibitor II and Mn, protein expression of full-length, C- and N-terminal fragments of alpha-synuclein were measured from brain homogenates. (A) Western blotting for full-length, C- and N-terminal fragments of alpha-synuclein and β-actin in the calpain inhibitor II and Mn-treated slices. (B) Semi-quantitative analysis of the protein expression of full-length alpha-synuclein. (C) Semi-quantitative analysis of the protein expression of C-terminal fragments of alpha-synuclein (C-terminal alpha-synuclein CCP Ab). (D) Semi-quantitative analysis of the protein expression of N-terminal fragments of alpha-synuclein (N-terminal alpha-synuclein CCP Ab). Expression of full-length, C- and N-terminal fragments of alpha-synuclein protein was normalized with β-actin protein. ** *P*<0.01 compared with control slices; ^#^ *P*<0.05 and ^# #^ *P*<0.01, compared with 400 μM Mn-treated slices.

Statistical comparisons revealed that there was a significant increase in the relative expression of alpha-synuclein mRNA after application of 400 μM Mn, compared with the controls (2.53-fold of control, *P*<0.01, Fig. 4), and pretreatment with calpain inhibitor II had no effect on the mRNA expression of alpha-synuclein compared with 400 μM Mn-treated slices. Similar to the increase in mRNA expression of alpha-synuclein produced by treatment with Mn, treatment with Mn also caused a significant increase in the protein expression of full-length alpha-synuclein compared with controls (4.10-fold of control, *P*<0.01, Fig. 5B). Strikingly, there was a significant increase in the protein expression of full-length alpha-synuclein in 4 μM calpain inhibitor II-pretreated slices compared with 400 μM Mn-treated slices (*P*<0.05). Pretreatment with calpain inhibitor II also caused a significant increase in the mRNA and protein expression of full-length alpha-synuclein when compared to the absolute controls.

### Cleavage of alpha-synuclein by calpain occurs in Mn-treated slices

To determine whether calpain cleaves alpha-synuclein in Mn-treated slices, we designed site-directed calpain-cleavage antibodies that specifically detect cleaved but not full-length alpha-synuclein using a method described previously [21]. Treatment with Mn also caused a significant increase in the number of C- and N-terminal fragments of alpha-synuclein compared with controls (6.38 and 3.99-fold of control, *P*<0.01, Fig. 5C, D). However, pretreatment with calpain inhibitor II caused a dose-dependent decrease in C- and N-terminal fragments of alpha-synuclein, with the maximum decrease in slices pretreated with 4 μM calpain inhibitor II (60.57% and 75.12%, respectively, *P*<0.01). These data suggested that calpain inhibitor II could inhibit the cleavage of alpha-synuclein.

**Fig 6:**
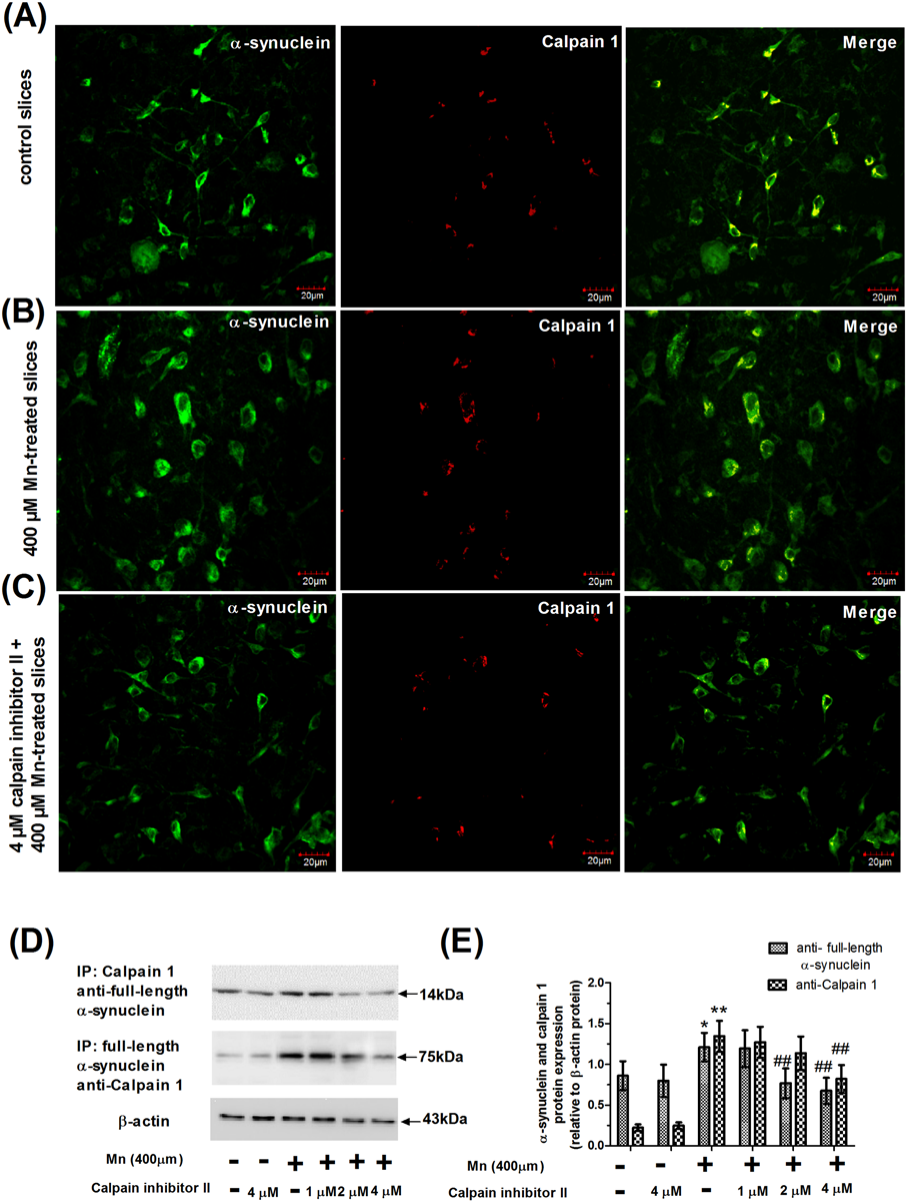
Calpain inhibitor II disturbed the interaction between alpha-synuclein and calpain 1. Colocalization fluorescence signals of alpha-synuclein and calpain 1 on brain slices were examined with an Olympus confocal microscope using the 40× objective lens. (A) Confocal microscopy images of immunofluorescence staining for C-terminal fragments of alpha-synuclein (green) and calpain 1 (red) on the non-treated slices. (B) Confocal microscopy images of immunofluorescence staining for C-terminal fragments of alpha-synuclein (green) and calpain 1 (red) on the 400 μM Mn-treated slices. (C) Confocal microscopy images of immunofluorescence staining for C-terminal fragments of alpha-synuclein (green) and calpain 1 (red) on the 4 μM calpain inhibitor II-pretreated slices. (D) The immunoprecipitation products of full-length alpha-synuclein and calpain 1 in the calpain inhibitor II and Mn-treated slices using co-immunoprecipitation. E) Semi-quantitative analysis of calpain 1 or alpha-synuclein. Expression of protein was normalized with β-actin protein. * *P*<0.05 and ** *P*<0.01 compared with control slices; ^# #^ *P*<0.01 compared with 400 μM Mn-treated slices.

We next investigated whether calpain 1 was involved in the cleavage of alpha-synuclein in situ using confocal laser scanning microscopy. Moreover, we also investigated the interaction of calpain 1 with full-length alpha-synuclein as well as the effect of calpain inhibitor II on the interaction of calpain 1 with full-length alpha-synuclein in Mn-treated slices. First, confocal microscopy was used to visualize the colocalization of calpain 1 and C-terminal fragments of alpha-synuclein in the nerve cell. For this purpose, brain slices were stained immunochemically with mouse anti-calpain 1 monoclonal antibody conjugated with Alexa Fluor 594. To detect C-terminal fragments of alpha-synuclein, the same slices were also incubated with rabbit anti-C-terminal fragments of alpha-synuclein polyclonal antibody conjugated with Alexa Fluor 488. Fig. 6 panels A, B, and C showed that both proteins were present in the cytoplasm of nerve cells, and as shown in the overlay images. Moreover, treatment with Mn also caused increase in the number of C-terminal fragments of alpha-synuclein compared with controls. However, pretreatment with calpain inhibitor II caused decrease in the number of C-terminal fragments of alpha-synuclein compared with only the Mn-treated slices. We next turned to co-immunoprecipitation experiments to detect a direct association of these proteins in nerve cells. To identify the interaction of calpain 1 and full-length alpha-synuclein, the brain slice lysates were incubated with either monoclonal alpha-synuclein antibody or polyclonal calpain 1 antibody. Immunoprecipitates were then isolated using a 50% slurry of protein A/G—agarose overnight at 4°C. Specifically bound proteins were solubilized in 40 μl of Laemmli sample buffer and separated by SDS/PAGE under reducing conditions using a 10% running gel. Both proteins were identified by immunoblotting of immunoprecipitates using specific antibodies (Fig. 6D, E). Thus, the interaction of calpain 1 and full-length alpha-synuclein was evidenced by: (a) blotting with anti-full-length alpha-synuclein, when the immunoprecipitate had been pulled down with antibody to calpain 1; (b) blotting with antibodies to calpain 1, when the immunoprecipitate had been pulled down with antibody to full-length alpha-synuclein (Fig. 6D). Semi-quantitative analysis showed that the interaction of calpain 1 and full-length alpha-synuclein decreased significantly and dose-dependently in response to calpain inhibitor II (1–4 μM), with the maximum decrease in slices pretreated with 4 μM calpain inhibitor II (*P*<0.01, Fig. 6E). Strikingly, Calpain inhibitor II pretreatment caused their affinity to decrease significantly in a dose-dependent manner, with the maximum decrease in slices pretreated with 4 μM calpain inhibitor II-pretreated slices (*P*<0.01, Fig. 6E), which was consistent with the notion that calpain inhibitor II disturbed the interaction between calpain 1 and full-length alpha-synuclein that results in the cleavage reduction of alpha-synuclein, but the interaction was still higher than those in the absolute controls.

### Calpain inhibitor II ameliorated Mn-induced alpha-synuclein oligomerization

**Fig 7:**
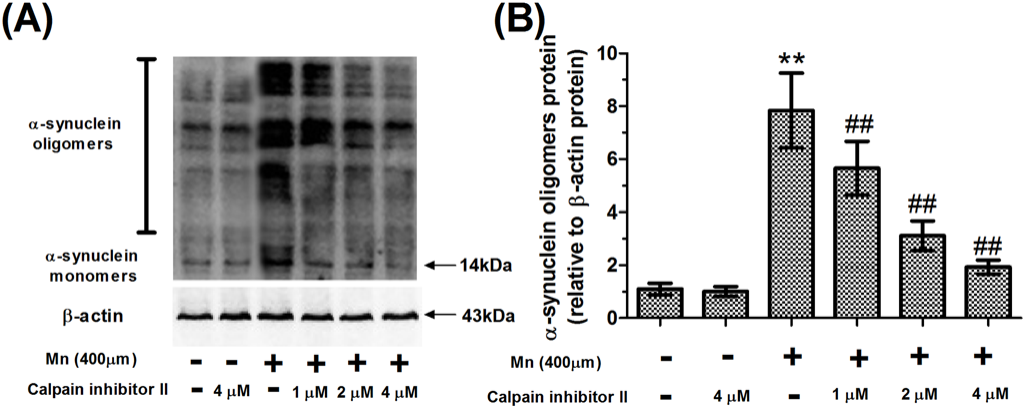
Calpain inhibitor II ameliorated Mn-induced the alpha-synuclein oligomerization in brain slice. After treatment with calpain inhibitor II and Mn for 24 hours, brain slices were homogenized. Total proteins were extracted and separated using 4–20% nondenaturing polyacrylamide gradient gel electrophoresis and analyzed using immunoblotting. (A) Western blotting for oligomeric/monomeric alpha-synuclein (full-length alpha-synuclein antibody) and β-actin in the calpain inhibitor II and Mn-treated slices. (B) Semi-quantitative analyses of the expression of oligomeric alpha-synuclein using image analyzing software (FluorChem v2.0) after western blotting experiments. Expression of protein was normalized with β-actin protein. ** *P*<0.01 compared with control slices; ^# #^ *P*<0.01 compared with 400 μM Mn-treated slices.

To verify that the alpha-synuclein oligomerization is associated with the cleavage of alpha-synuclein, we analyzed oligomeric alpha-synuclein of calpain inhibitor II- and Mn-treated slices using 4–20% nondenaturing polyacrylamide gradient gel electrophoresis and immunoblotting (Fig. 7A). Treatment with Mn resulted in a significant increase in alpha-synuclein oligomers (*P*<0.01, Fig. 7B). Strikingly, alpha-synuclein oligomers were reduced by pretreatment with calpain inhibitor II because of its inhibition of calpain activity, but the alpha-synuclein oligomers in 1 and 2μM calpain inhibitor II-pretreated slices were still higher than those in the absolute controls(*P*<0.01, Fig. 7B). Semi-quantitative analyses of the expression of oligomeric alpha-synuclein found that their changes in expression were consistent with the changes in cleavage fragments of alpha-synuclein. These results suggested that the cleavage of alpha-synuclein by calpain was an important signaling event in the Mn-induced alpha-synuclein oligomerization.

## Discussion

Neurodegenerative diseases are increasingly prevalent in our communities recently due to several causes such as aging, heritage and heavy metals exposures. Mn is one of the heavy metals causing neurodegenerative dysfunction that is similar to but somewhat different from PD [32]. The mechanism of cell death induced by Mn toxicity has not been understood clearly yet although several hypotheses have been suggested. We used an in vitro brain slice culture model to study Mn neurotoxicity as the brain slices offer distinct advantages and bypass complications arising from the presence of the blood—brain barrier, peripheral metabolism, and other peripheral effects known to occur in vivo. In the current study, we made two important observations, the first of which was that cleavage of alpha-synuclein by calpain occurs in Mn-treated slices. The second was that calpain-cleaved alpha-synuclein fragments could promote alpha-synuclein oligomerization and enhance cellular toxicity.

In this study, brain slice injury was assessed by the release of LDH and apoptosis assays, which are reliable and reproducible parameters to assess the extent of neurocyte damage by Mn-treatment. LDH that is normally retained within the cell was released into the medium from damaged neurocytes. LDH release is an indicator of the integrity of the cell membrane. Treatment with Mn resulted in a significant increase in levels of LDH in the culture medium. Moreover, to further confirm irreversible neurodegeneration and facilitate quantification following manganism, neurocyte apoptosis was assessed by flow cytometry. The percentage of apoptotic cells from the control slices might be an outcome of spontaneous and rapid apoptosis of dissociated slice cells. Furthermore, our findings demonstrated that exposure to 400 μM Mn caused the apoptotic percentage to increase up to 29.6%. These results indicated that Mn treatment caused appreciable neurotoxicity. As previously reported [9], we consider the mechanism of Mn-induced neurotoxicity to be related to alpha-synuclein oligomerization, which was supported by our data.

Ca^2+^ homeostasis is critical for regulation of cellular function. Ca^2+^ homeostasis dysregulation will ultimately lead to neuronal degeneration through massive activation of cellular proteases such as calpains [33]. Under physiological conditions, calpains exist as inactive proenzymes in the cytosol. Once they are activated by increased cytosolic Ca^2+^ load, calpains degrade a large number of cellular proteins, including cytoskeletal proteins and alpha-synuclein protein ultimately leading to neurodegeneration and neurological dysfunction. Our previous studies have found that overactivation of N-methyl-D-aspartate receptors (NMDARs), a phenomenon known as excitotoxicity, by Mn could result in an influx of extracellular Ca^2+^, which triggers a series of toxic events, ultimately leading to cell death [24]. In this study, in vitro treatment of brain slices with Mn for 24 hours resulted in a significant increase in [Ca^2+^]_i_ and the activity, mRNA expression, and protein expression of calpain. The results showed that calpain activity was excessively activated by [Ca^2+^]_i_. However, it has also been demonstrated that calpain inhibitor II could inhibit the activity of calpain and reduce Mn-induced nerve cell injury. Our finding was consistent with previous research concerning the protective effect of calpain inhibitor II on nerve cell injury [20]. To confirm that calpain inhibitor II is non-toxic in low doses, we designed the present study to include a calpain inhibitor II alone group. The results demonstrated that there were no statistically significant differences in brain slices that were treated with calpain inhibitor II alone compared with controls. Based on these results, we concluded that low doses of calpain inhibitor II not only could inhibit the pathological consequences of calpain overactivation but also preserve physiologic aspects of calpain function.

Mishizen-Eberz et al. [19] found that the conspicuous presence of lower molecular mass alpha-synuclein species in alpha-synuclein aggregates and the enhanced in vitro fibril assembly of recombinant C-terminally truncated alpha-synuclein suggests that the low-molecular mass alpha-synuclein species may be of pathogenic significance. The cleavage of alpha-synuclein at either the N- or C-terminal end of alpha-synuclein could be detected in Mn-treated slices using two site-directed calpain cleavage antibodies. In the present study, treatment with Mn caused a significant increase in the number of C- and N-terminal fragments of alpha-synuclein. Strikingly, there was a significant increase in the protein expression of full-length alpha-synuclein and decrease in C- and N-terminal fragments of alpha-synuclein in 4 μM calpain inhibitor II-pretreated slices, which could be explained by inhibited calpain activity. To further confirm the cleavage of alpha-synuclein associated with calpains, we employed confocal laser scanning microscopy and co-immunoprecipitation assays. The data provided direct evidence for the interaction between alpha-synuclein and calpain 1 in neuronal cells. We demonstrated that calpain 1 interacted and colocalized with alpha-synuclein in the cytoplasm of neuronal cells. Therefore, calpain 1 formed a complex with alpha-synuclein protein and promoted the cleavage of alpha-synuclein under pathological conditions. Our results also showed that treatment with Mn resulted in a significant increase in alpha-synuclein oligomers, and alpha-synuclein oligomers were reduced by pretreatment of calpain inhibitor II, which were consistent with the changes of cleavage of alpha-synuclein. Based on these results, we concluded that the overactivation of calpains was one of the important reasons leading to alpha-synuclein oligomerization.

## Conclusions

Taken together, the results of this study showed that Mn induced the cleavage of alpha-synuclein protein via overactivation of calpain 1 and subsequent alpha-synuclein oligomerization in cultured slices. Moreover, inhibition of calpain could partially inhibit Mn-induced nerve cell injury. Our results give insight into the neurochemical alterations that take place in nerve cells during elevated Mn exposure, and inhibition of calpain may represent a novel therapeutic target to ameliorate neuronal damage in manganism.

## Supporting Information

S1 Fig Dissociated basal ganglia cells were analyzed using flow cytometry. The Q4 quadrant was considered to contain the early apoptotic cells (FITC＋/PI－); a: control slices, b: 4 μM calpain inhibitor II, c: 400 μM Mn slices, d: 400 μM Mn +1 μM calpain inhibitor II, e: 400 μM Mn + 2 μM calpain inhibitor II, f: 400 μM Mn + 4 μM calpain inhibitor II.
(TIF)
