# Supplementary figures and images for: Inhibition of Calpain Prevents Manganese-Induced Cell Injury and Alpha-Synuclein Oligomerization in Organotypic Brain Slice Cultures
Source: PLoS One. 2015 Mar 10;10(3):e0119205. doi: 10.1371/journal.pone.0119205 (PMC4355489; doi:10.1371/journal.pone.0119205)

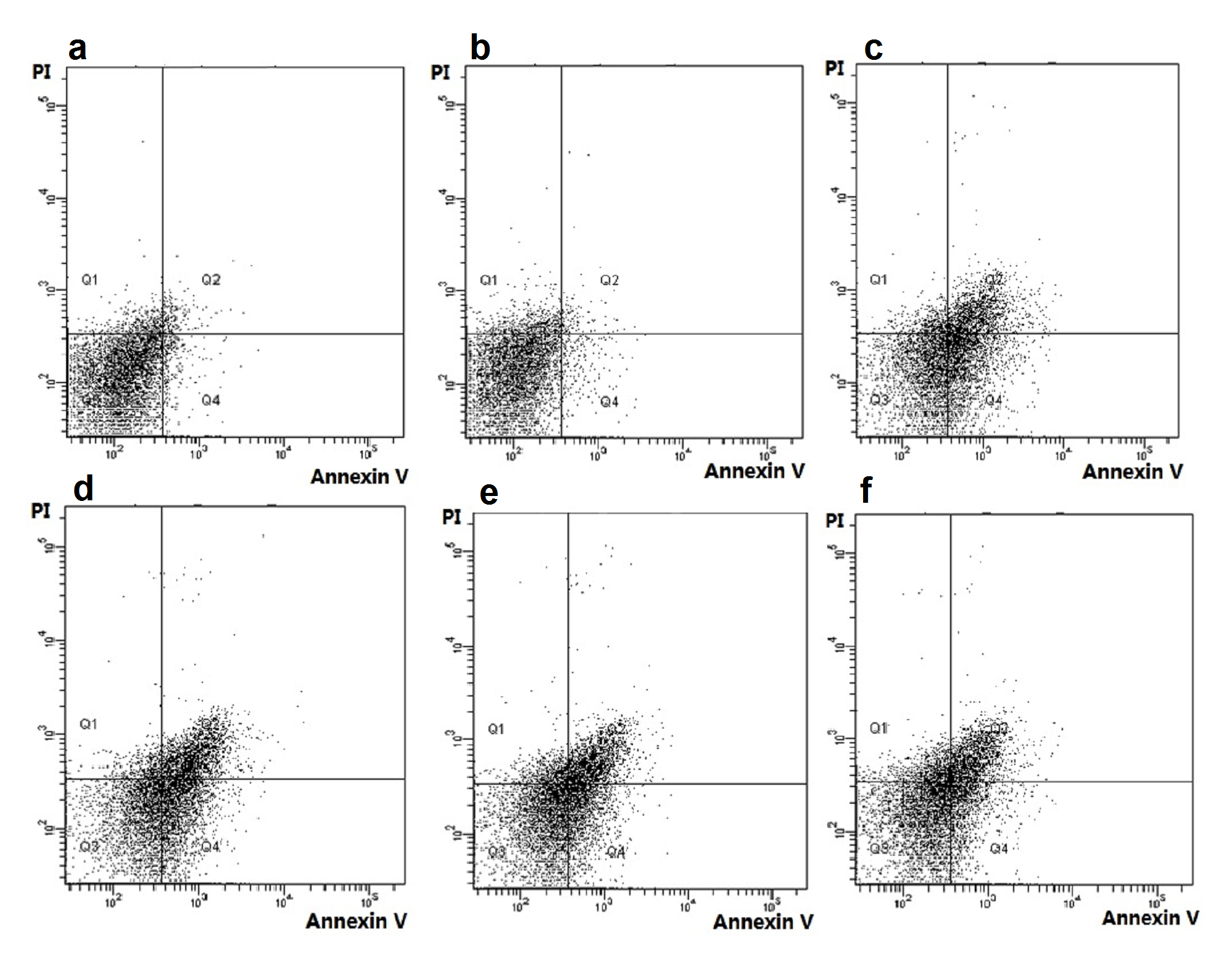

Supplement: S1 Fig — The Q4 quadrant was considered to contain the early apoptotic cells (FITC＋/PI－); a: control slices, b: 4 μM calpain inhibitor II, c: 400 μM Mn slices, d: 400 μM Mn +1 μM calpain inhibitor II, e: 400 μM Mn + 2 μM calpain inhibitor II, f: 400 μM Mn + 4 μM calpain inhibitor II. (TIF) [file pone.0119205.s001.tif]
